# Accessibility of cancer treatment services for Indigenous Australians in the Northern Territory: perspectives of patients and care providers

**DOI:** 10.1186/s12913-021-06066-3

**Published:** 2021-01-28

**Authors:** Kate Anderson, Abbey Diaz, Darshit Rajeshkumar Parikh, Gail Garvey

**Affiliations:** 1 Menzies School of Health Research, Wellbeing and Preventable Chronic Diseases Division, Charles Darwin University, Darwin, Northern Territory Australia; 2 School of Psychological and Clinical Sciences, Charles Darwin University, Darwin, Northern Territory Australia

**Keywords:** Cancer, Aboriginal and Torres Strait islander Australians, Cancer treatment, Health services, Access, Equity, Patient-centred care

## Abstract

**Background:**

Poorer cancer outcomes of Indigenous Australians in Australia’s Northern Territory (NT) compared with their non-Indigenous counterparts are partially due to diminished access to cancer treatment services (CTS). Accessibility of health care is a multidimensional construct, including physical, logistical, psychosocial and cultural dimensions. While previous research has identified specific areas of reduced access to CTS for Indigenous Australians, the higher burden of cancer borne by Indigenous Australians warrants a more comprehensive understanding of access to CTS in the NT. The purpose of this study was to explore and map the accessibility of CTS for Indigenous Australians in the NT and to identify key access barriers.

**Methods:**

This predominantly qualitative study, complemented by a descriptive quantitative component, explored and mapped the accessibility of one CTS (CTS-NT) that services a large number of Indigenous Australians in the NT. Patient perspectives were obtained via secondary analysis of data from 75 face-to-face interviews with Indigenous Australian adults attending the CTS-NT. Care provider perspectives were obtained via primary analysis of data from 29 face-to-face interviews with care providers and staff working at CTS-NT. Data were analysed to identify issues of accessibility informed by Leveque and colleagues’ conceptual framework of access to health care, which comprises five dimensions of *accessibility* of the health service and the *ability* of Indigenous patients to interact with these dimensions to generate access. Applied thematic analysis was conducted on the qualitative data and descriptive analysis was conducted on the quantitative data.

**Results:**

The analysis of the patient and care provider reports identified multiple access barriers across all dimensions including: inadequate preparation of Indigenous patients for treatment; delayed and complicated commencement of treatment; dislocation from home; competing priorities; scarcity of Indigenous care providers and staff; lack of culturally-relevant care; challenges associated with language, accommodation, transport and finance; and disjointed and fraught relationships with care providers. These barriers posed significant challenges to Indigenous patients maintaining their engagement with treatment.

**Conclusions:**

This study provides a valuable snapshot of the barriers facing this population across the dimensions of health care access. Urgent action in addressing these issues is required at individual, service and state levels.

## Background

The Northern Territory (NT) is a large territory in the central northern region of Australia and is the least-populous of Australia’s eight states and territories. The proportion of people in NT who identify as Indigenous (32%) is much higher than for other states and territories [1]. People living in remote areas of Australia are often disadvantaged in relation to access to primary health-care services, educational and employment opportunities, and income [2]. The burden of cancer for people living in the NT is substantial, with a higher overall cancer mortality rate than any other Australian jurisdiction [2–4]. Moreover within the NT, the mortality rate for all cancers combined was significantly higher for Indigenous Territorians compared to non-Indigenous Territorians (340 deaths and 184 per 100,000 respectively) [5]. In addition, the cancer hospitalization rate between 2008 and 2012, was lower for Indigenous compared to non-Indigenous Territorians (10 per 1000 compared with 14 per 1000) [5]. There is growing evidence that these disparities may be partially due to reduced access and engagement with cancer services [6, 7]. Indigenous Australians are less likely to access cancer screening [8]; have relatively fewer cancer-related hospitalisations [9]; are diagnosed at a later cancer stages [3, 9, 10]; and are less likely to receive cancer treatment compared to other Australians for the same primary site, age and diagnostic periods [11, 12].

While the notion of *access to health care* has been variously defined, Levesque and colleagues offer a useful conceptual framework for identifying the components and determinants of access to health care [13]. They define *access* as ‘the opportunity to have health care needs fulfilled’ [13], and their framework distinguishes five dimensions of accessibility of health services: 1) approachability; 2) acceptability; 3) availability and accommodation; 4) affordability; and 5) appropriateness [13]. This framework provides a practical model to consider the accessibility of CTS for Indigenous Australians and identify critical barriers to access and solutions to overcome them.

The following factors have been identified over the past two decades as affecting Indigenous Australians access to CTS: difficulties navigating the health system; low health literacy; logistical impediments; remote living location; gaps in care between primary and tertiary health services [14–16]; a high burden of comorbidity [17]; a historically-based wariness of government organisations [18]; fatalistic perceptions of cancer [3, 19]; differing health paradigms [20]; cultural diversity; and language differences [15, 21]. The extent to which CTS have adapted to overcome these and other barriers to improve access for Indigenous people is unclear and further research is needed to better understand and respond to issues of accessibility to CTS, particularly in more remote locations where people are likely to face multiple challenges.

The geographic and population characteristics of the NT present a wide range of challenges to accessing CTS for Indigenous Australians. These include but are not limited to: a large and seasonally inaccessible geographic area; very large distances between CTS locations; many of the NT’s Indigenous population live in remote and very remote communities; a large number of diverse Indigenous languages and cultural groups; and a largely transient health workforce in the NT [22]. These factors make the NT an expedient location for exploring a likely broad range of accessibility issues, many of which will have relevance for other regional and remote locations in Australia and globally.

The aim of this study is to explore the accessibility of a major hospital-based CTS in the NT for Indigenous patients. The self-reported views of Indigenous Australian adults receiving cancer treatment and care providers and staff working at the CTS-NT were analysed to identify issues associated with Indigenous people’s access to this service.

## Methods

### Participants, setting and data collection

This study used a predominantly qualitative approach, complemented by some quantitative survey questions, conducted at CTS-NT, which annually treats around 800 cancer patients, 30% of whom identify as Indigenous Australian. Data from interviews conducted with patients and care providers have been included in this study to enable a comprehensive analysis including multiple perspectives on the accessibility of CTS-NT for Indigenous patients.

#### Patient interviews

The patient data reported in this study comes from a larger dataset collected from participants in a multi-centre, cross-sectional project conducted over a 12 month period in 2014–2015 that investigated the supportive care needs of Indigenous Australian cancer patients [23]. Patients at the CTS-NT were asked additional questions related to CTS accessibility and these participants are included in the current analysis. The detailed methods for the larger study are published elsewhere [23]; in brief, study participants were Indigenous adults diagnosed with cancer within the past 5 years and who were attending CTS-NT. Data was collected via face-to-face structured interviews conducted by trained interviewers and took approximately 45–60 min each. Participants were asked closed and open-ended questions relating to their experiences of and views regarding access to CTS-NT [see Appendix 1 – Questions asked of patients included in this analysis] and this information was recorded by the interviewer on the data collection form, who was an employee of the cancer care centre and, for the interviews, employed by the project as a researcher officer. Participants’ responses to open-ended questions were paraphrased by the interviewers at the time of interview. Patient self-reported socio-demographic information such as age, marital status, main language spoken at home, education level, employment status, and residential postcode and community/township was also collected.

#### Care provider interviews

Care providers from the CTS-NT were purposively sampled to include perspectives from a diversity of roles across the health care team [see Appendix 2 – Questions asked of care providers included in this analysis]. Potential participants were informed about the study by the Radiation Oncology Manager of CTS-NT and were invited to be interviewed. Those interested were contacted by email or in person by the interviewers (KA, DP) to confirm their willingness to participate, and if so, gain verbal and written consent to be interviewed. Two researchers (KA, DP) conducted semi-structured, face-to-face qualitative interviews in November 2017 with cancer care providers including cancer specialists, nurses, allied health professionals and administrative staff from the cancer centre. The interviews, which ranged in duration from 9 min to 39 min, were audio-recorded, transcribed verbatim, checked for accuracy and de-identified for analysis.

### Data analysis

Qualitative and quantitative data were first analysed separately and then concurrently for the final interpretation and presentation of the results. Patient sociodemographic and clinical characteristics, and patients’ responses to closed questions (quantitative data) were entered in Microsoft Access and imported into Stata (Version 15) for descriptive data analysis. Frequencies and proportions were used to describe categorical data and continuous data were described using means and standard deviations (SD). Professional characteristics of care providers were summarised using Microsoft Excel 2018. The qualitative data from patient and care provider interviews were imported into NVivo11 [24] to facilitate an applied thematic analysis [25] and one researcher (KA) coded the data to identify themes and subthemes.

**Fig. 1:**
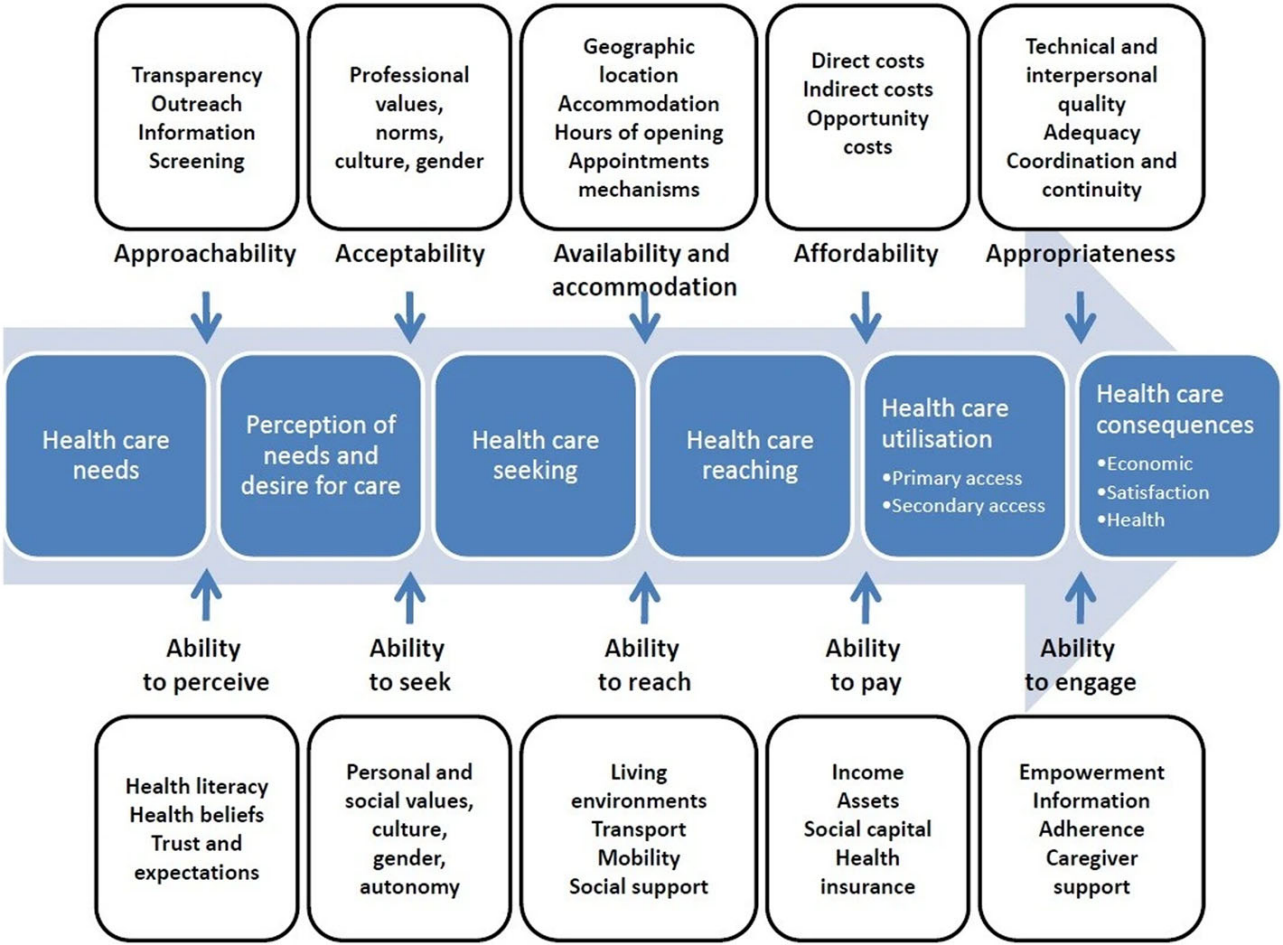
Levesque and colleagues’ conceptual framework of access to health care

Levesque and colleagues’ conceptual framework of access to health care [13] was used to guide the structure, interpretation and reporting of the coding and qualitative analysis of this data (see Fig. 1). The data from patients and care providers were coded separately, but analysis was undertaken and presented conjointly to enable mapping across the corresponding dimensions of *accessibility* of the health service and *ability* of individuals to interact with the dimensions of accessibility to generate access [13]. The views of patients and care providers are identified throughout the results.

## Results

A total of 75 Indigenous patients (45 female and 30 male) were interviewed. The majority of Indigenous patients were diagnosed with cancer within six months prior to the interview date (60%) and the most common cancer types were: breast cancer (23%), digestive system cancers (19%), head and neck cancer s (19%), and lung and other respiratory system cancers (17%). Most were not in paid employment (79%) and had at least one comorbid disease (72%). Slightly more patients were older than 50 years (56%) and reported not having a spouse (52%).

A total of 29 care providers (21 female and 8 male) were interviewed. Care providers held diverse roles, including Radiation/Medical Oncology Consultants (*n* = 5); Oncology Nursing Staff (n = 5); Radiation Therapists (*n* = 8); Indigenous Liaison Officer (*n* = 1); Cancer Care Coordinator (*n* = 2); other Allied Health professional (*n* = 3); managerial, administrative and support staff (*n* = 4); and a medical student (*n* = 1).

Qualitative analysis of the patient and care provider interviews revealed several key factors impacting Indigenous cancer patients’ access to CTS-NT across the five service-level dimensions and the corresponding patient-level abilities of the Levesque conceptual framework of access to health care. These barriers are described in detail here and are summarised in Table 1.

**Table 1:** Barriers facing Indigenous patients accessing CTS-NT across the five dimensions of accessibility

DIMENSION	BARRIERS FACING INDIGENOUS PATIENTS ACCESSING CTS-NT
**Approachability**	• Patients being ill-prepared and poorly-informed about their cancer
	• Patients being poorly-informed about the nature and justification for the treatment
	• Mistrust of mainstream health services
	• Delayed and complicated commencement of cancer treatment
	• Dislocation from home while accessing treatment
	• Juggling priorities at home with the demands of cancer treatment
**Acceptability**	• Scarcity of Indigenous care providers and staff at CTS-NT
	• Incongruity of values between Indigenous patients and the CTS-NT
	• Insufficiency of culturally-sensitive care
	• Challenges associated with language, translation, and communication
**Availability and accommodation**	• Difficulties accessing transport
	• Inappropriate and/or unacceptable accommodation and food
	• Dislocation from social support
**Affordability**	• ‘Hidden costs’ associated with travel, accommodation and food
	• Loss of income occasioned financial hardship
	• Challenges supporting family
	• Uncertainty around financial supports available to patients
	• Lack of knowledge on where access information about financial support
**Appropriateness**	• Disjointed and fraught relationships with care providers
	• High staff turnover rates hindering culturally-sensitive care

### Approachability

The *approachability* dimension of access refers to the degree to which people with a health need can identify that a suitable service exists, can be reached, and will benefit them [13]. While our analysis focuses on people who have some level of engagement with CTS-NT, the participant views reveal considerable approachability-related barriers to Indigenous people initiating and maintaining this engagement – particularly for those living outside of CTS-NT’s regional location.

Care providers also spoke about Indigenous patients commonly arriving at CTS-NT unaware that they had been diagnosed with cancer. It was suggested that staff in community health settings were reluctant to explicitly inform Indigenous patients of their cancer diagnosis, instead using nebulous terms like ‘biopsy’, ‘test’ and ‘treatment’ to explain the patient’s need to go to Darwin for medical treatment. Care providers regarded this unpreparedness as occasioning shock and mistrust for many Indigenous patients. This may have undermined patients’ confidence in the arduous and lengthy treatment regimen, which already involved enormous upheaval and strain for patients.

*‘Because when* [patients] *come here, obviously you have patients that are not well prepared and uninformed, some of them don’t even know they have got cancer, so, it is the early part of the journey that is the issue in the Northern Territory in the sense that, you know, patients get told, “you need to do this test, you need to do this biopsy,” and then sometimes the information about informing the patient that they got cancer is missed.’ [Care provider, CP003a].*

While mistrust of mainstream health services may deter Indigenous cancer patients from engaging with services, it may also impact on their perceived acceptability of these services. Therefore, this finding is also relevant for acceptability, according to the Levesque Framework [13].

While not a common theme among the patient participants, one patient suggested that to overcome dis- and mis-information about cancer diagnosis and treatment, Traditional Healers could be invited to the CTS-NT to undertake a cancer education training program so that they could better explain to community members who are diagnosed with cancer about the nature of their condition and treatment and the benefits of maintaining their treatment regimens.

Care providers also described that timely diagnosis and commencement of treatment was a key challenge, as many Indigenous patients present for treatment in advanced stages of cancer. This was attributed by care providers to multiple causes, including: limited outreach services; variable quality of communication with community health services; limited access to prevention and screening programs; the lack of identification of symptoms in early cancer stages; a reluctance to seek treatment due to an association between cancer treatment and death; and an historically-based mistrust of Western medical services. Care providers identified these issues as barriers to Indigenous patients seeking medical help, commencing cancer treatment and maintaining their cancer treatment once they had started.

*“*[A patient] *might be “lost to follow up” or “lost” because they have gone off the radar or gone out bush, and then that has delayed their treatment time and then that has obviously played a big impact on their outcome, and then there is other times where they won’t realise the severity of their diagnoses.”[Care provider, CP002].*

The delayed and complicated start of cancer treatment for many Indigenous patients was often associated with ongoing challenges in maintaining treatment regimens. Approximately one quarter of patients reported missing appointments since their treatment commenced. Patients explained their reasons for missing appointments, included: needing to return home for Sorry Business,^1^ worrying about situations in their home community, transport problems, issues with communication from CTS-NT staff, hostel staff and transport providers, and pressure from patients’ escorts or carers wanting to return home. For example, one patient described encountering ‘*problems when the escort wants to go home. Not everyone can stay for the duration of treatment.’ [Patient interview].*

Care providers also spoke about Indigenous patients disengaging with treatment and missing appointments, which was attributed to transport difficulties, miscommunication between care providers and patients, patients returning to Community for Sorry Business and having a different conception and/or priority of time to the service providers.

*‘Appointment schedules confusing. Process confusing. Just a sheet of times and dates. Need to sit down with nurse or* [Indigenous Liaison Officer] *and explain.’ [Patient interview].*

Several care providers described how the CTS-NT had modified their service to offer greater flexibility, particularly around appointments, and individualised cancer care in order to minimise the access barriers for patients.

*“I think [*Indigenous patients*] don’t have a time awareness so they don’t come on time. So, it’s a bit challenging to the health system because we are booked back to back and they don’t come on time, but they don’t know time, so it’s not their fault.” [Care provider, CP010].*

*“We’ve just become accustomed to … we have transport issues, so the patients might come on buses that may not always run to schedule. We’ve just learnt to be flexible.” [Care provider, CP004].*

The approachability related barriers served to delay, discourage and challenge Indigenous people from engaging with their cancer care. Several patients expressed confusion and uncertainty about what the treatment process involved, and some patients expressed feeling inadequately informed about their health condition.

### Acceptability

The *acceptability* dimension relates to cultural and social acceptability of the service to those seeking care, including the ethnicity, sex or social group of care providers and the health belief systems underpinning the service [13]. The patients and care providers highlighted critical barriers in this area which centred around the scarcity of Indigenous staff working at the CTS-NT and the incongruity between the values underpinning the CTS-NT service and those held by Indigenous people.

The need to increase the number of Indigenous staff members, particularly those in clinical roles, was flagged by several care providers. It was also suggested that the CTS-NT would benefit from engaging an Indigenous spiritual healer to provide traditional medicine options to their patients.

The Indigenous Liaison officer (ILO) was described by many care providers as the main means of identifying the language and cultural requirements of Indigenous patients. The ILO was the link between the doctors and the patients to enable individualised, patient-centred care for this patient group. All care providers stressed the invaluable role of the ILO in bridging the, often vast, gap between medical services and Indigenous patients. Several respondents suggested the need for more than one ILO at the CTS-NT.

Patients also alluded to the importance of the ILO’s role, particularly in supporting communication and cultural mediation between patients and care providers.

*‘*[The ILO] *helps me to understand what the doctors are telling me. I need her to help because I don’t have escort with me.’ [Patient interview].*

Despite the clear value of the ILO, there was only one ILO employed at the CTS-NT, and their position was not filled when they were absent on leave or working elsewhere. This situation was described by the ILO as leaving the Indigenous patients at a significant disadvantage and them feeling a sense of guilt for going on leave.

Care providers identified a mismatch between the mainstream biomedical paradigm of CTS-NT’s services and the holistic views of health they ascribed to Indigenous patients.

*“The key thing about Indigenous patients in the pathways is their priorities are not the same as non- Indigenous patients, obviously. They have got family commitments and so forth, and all the other communities. So, in the model of care that we expect non-Indigenous patients – where they drop everything, and cancer becomes the prime focus – it may not be the same for Indigenous people. So, we have to, in a way respect that and get them to participate. We may not be sure that they are well-informed to all the seriousness of their cancers, because their concept of life and living and so on, it is a little bit different.” [Care provider, CP003a].*

The diversity of Indigenous people’s culture and language across the Northern Territory was put forward by care providers as a challenge to integrate into the ‘*one size fits all’* biomedically-based treatment setting. Care providers reported there were many Indigenous dialects in the Northern Territory, and the efficacy and availability of interpreter services was variably effective. This in turn put inappropriate pressure on the ILO to act as a translator on some occasions. English was often said to be the fifth or sixth language for many remote-living patients, which makes effective clinical communication more complicated. As there are no direct translations for most medical terms into Indigenous languages, care providers explained that they often use metaphors to convey their clinical message. Longer appointments are required for Indigenous patients to ensure they understood what was happening and being communicated.

*“Because one bloke [a* patient*] said to me, he goes, “You have to understand, sister, I’m translating to four languages before I get back to you.” So, he found it difficult to go from English to his language, he had to go to another language, sort of, into another one, and then back to his and then go, okay, this is what it means, and then come back. So, a 15-min consult took an hour and a half.” [Care provider, CP020].*

### Availability and accommodation

The *availability and accommodation* dimension refers to if the health service can be reached, physically and in a timely manner [13]. All study participants reported that many Indigenous patients experience substantial access challenges associated with transport, accommodation, food and social support.

The patient reports revealed that 65 patients (87%) had to move away from home to receive cancer care. Those patients who had relocated from their home for treatment had been in Darwin for time periods ranging from 1 day to 6 months, with about half being away from home between one and four weeks. Of the 65 Indigenous patients who relocated to receive their cancer care, almost all (98%) had to do so due to the lack of treatment services closer to home.

All of the patients who relocated had heard of and used the Patient Assistant Travel Scheme (PATS) to subside the cost of their travel to seek treatment. Many patients reported difficulties in using this scheme, it was described as an inflexible system with a confusing booking system. Transportation was an issue as buses were often not on time, or they forgot to collect patients. Accommodation bookings were often hostel rooms that did not accommodate patients with wheelchairs or their escorts.

*Patient has been living with son in Darwin for extended time due to treatment. Cannot get assistance to travel back to community. [Patient interview].*

Many patients walked to the supermarket from their accommodation to buy groceries, while others reported using the bus, taxi, or getting a ride with family or friends. The taxi was often preferred; as one participant explained, this was due to the bus station being too far away and feeling unsafe walking at night. However, due to the cost, taking a taxi was not always viable.

The majority of patients who travelled away for their cancer care or treatment were supported with accommodation during this time, but over a third reported difficulties with accommodation. The difficulties around accommodation described by patients included: unsuitability of accommodation and food, and a lack of accommodation for family and/or escort. Most patients reported that they needed their escort at the accommodation. Some patients described transient living arrangements, moving between staying with relatives and *long grassing.*^2^ One such patient, who had missed multiple appointments, described the strain on her health she experienced during long grassing over the dry season.

However, another female patient expressed a preference for living in the long grass. She explained that living in the hostel made her feel disconnected from her culture, friends and Country. She felt more supported in the long grass and can forget her worries when surrounded by familiar sounds, smells, friends and stories.

Several patients explained that the hostel accommodation in Darwin is unsuitable for Indigenous people, especially those with family and escorts with young children. Moreover, hostel accommodation was said to be far from shops and to feel more like a prison than lodging.

*‘You cannot get any rest there. It is frightening.’ [Patient interview].*

Most people had someone go to their appointments with them (68%) and for the majority of these patients this role was fulfilled by the escort (86%). Partners (24%) and other family members (57%) commonly fulfilled the escort role for the patients. While escorts often came with patients from out of town, the reliability of escorts was reported by care providers to be varied. This meant that patients were said to be sometimes left unsupported. Care providers said that the ILO was constantly assessing and reassessing the needs of Indigenous patients in relation to their travel, accommodation, financial situation and the status of their escort or support person.

*“In cancer, you have to accommodate and treat everything. The full spectrum, from psycho-social family issues*. *.. because they will impact on the cancer journey because the cancer journey is so vast and complex. .. without attending to any of those* [additional issues]*, you know, you tend to have breakdown in compliance and so forth.” [Care provider, CP003a].*

Other care providers also highlighted the view that Indigenous patients who relocate from remote communities to access treatment experience a range of stressors stemming from the move. Care providers felt that staying in Darwin was daunting and stressful for Indigenous patients, who often had little previous experience of a city. Navigating hostel accommodation, unfamiliar food and inflexible transport were all identified as issues reported to care providers by patients. Additionally, care providers reported that some Indigenous patients disengaged with treatment prematurely to return to their community due to the stress associated with being away from their own family and community responsibilities.

*“I think separation from family is a big thing for [*Indigenous patients*] and accommodation, and again, it’s a major thing for them because although we are accommodating them, all our current accommodations are, the majority of them are air-conditioned accommodation and they serve Western food, they allow only one person to stay with the patient; they restrict visitors. That’s not how their social situation is, and they immediately feel claustrophobic and they feel isolated.” [Care provider, CP010].*

Care providers described offering patients telehealth as a means to overcome these difficulties and reduce the time patients need to stay in town. Similarly, while patients are at CTS-NT, some care providers reported offering them opportunities to connect with community and family via video conference. This was described as important in facilitating community decision-making about the patient’s treatment, as well as reducing the patients’ isolation from family.

*“Most of my first consults have 20 people in the consult.*. *.Before I start my first consult, I will see the referral, I will go and talk to my ILO and would say, okay, can you find out who will need to be in the consult … so she will go and find out all the names of the people who want to be in the consult. So, we dial up, we have tele link with the communities and we have extended family, 20, 30 people, it will take a lot of time but often I have left the patient with family at the end of the consult, to have a conversation amongst themselves and I come out from the room so that they are comfortable.* “*[Care provider, CP010].*

### Affordability

The *affordability* dimension reflects people’s ability to spend the required money and time to use appropriate services. While many financial costs associated with accessing treatment were not bourn directly by patients, numerous ‘hidden’ costs were evident in the reports of patients and care providers that considerably impacted on treatment access.

Patients reported being poorly-informed around what financial supports they were entitled to, and how to find out about what was available to them. Many Indigenous patients had left jobs, family and financial responsibilities back in their home communities, and were having to find money for food and to pay additional expenses associated with life in town. Several patients expressed confusion about payments and reimbursements for travel, with some people being left significantly out of pocket due to miscommunication around receipts and processes.

*‘PATS-not reimbursing in a timely manner. Still waiting on reimbursement for travel ... This is the worst thing. General living expenses are now very hard.’ [Patient interview].*

*The travel system is not fully explained. Patient was not advised that she could be reimbursed for taxi when bus schedule didn’t meet her needs. She did not keep her receipts so couldn’t claim fare back. [Patient interview].*

Patients also reported difficulties in dealing with Centrelink (Australian Government payments and services) to access financial support due to loss of employment, illness and not being able to provide proof of their usual place of residence when they were highly transient.

A few care providers were cognisant of the financial pressures and other responsibilities on Indigenous patients who relocated to receive cancer care.

*‘When [*Indigenous patients*] come in here sometimes they’re concerned that they don’t have any money, and my job is to reassure them that their accommodation and their meals are paid for. That sort of thing. But their problem is, it’s not so much their needs, it’s family’s needs for, and that sort of thing. So, family needs back home as well, with them being here.’ [Care provider, CP001].*

*‘[*Indigenous*] people are coming away from their communities – they have money issues, family issues, job issues, you name it. Let alone dealing with their cancer.’ [Care provider, CP022].*

### Appropriateness

The *appropriateness* dimension denotes the fit between service offerings and patient needs, including the interpersonal quality of the care, suitability of the setting and care provision, and the service’s ability to empower patients to actively engage with treatment [13].

Almost half of the patients reported feeling lonely since their cancer diagnosis. However, the majority reported that they had someone to confide in and share feelings with about their cancer, with half of these reporting that they talk to this person daily.

Some patients expressed difficulties in their relationships with doctors’ and nurses’, including dissatisfaction with the way care providers had communicated information with them in a culturally unsafe way.

*‘Balanda*^3^*just doesn’t understand how it is with us. .. How frightening it is at the hospital, and meeting doctors, especially when they don’t have an escort with them.’ [Patient interview].*

*‘Doctor and nurse speak to me in a pessimistic manner. Need more positive feedback. “End of Life” discussion, brought up by oncologist, was not appropriate for Aboriginal patients.’ [Patient interview].*

Care providers commonly reported having difficulty identifying when and how to provide emotional support for Indigenous patients. There is limited social work presence at the CTS-NT and Indigenous patients are rarely referred to counselling or psychology services as there was some uncertainty raised by care providers about the appropriateness of these services for Indigenous people. Social assistance and support for this patient group is usually channelled through the ILO. Several care providers expressed the views that Indigenous patients avoid talking about their cancer and would not be open to joining cancer support groups. However, it was suggested by one allied health provider that a more informal method of sharing stories and support might be beneficial for Indigenous patients.

*‘ … having some sort of social network where we could support them, … like, a barbecue in the ground or, like, a little billy tea bushfire or something like that. Something that they would normally do back home that they could do here, that would just make them feel that little bit more integrated.’ [Care provider, CP020].*

Care providers regarded the high CTS-NT staff turnover rate as a challenge for building rapport and trust with Indigenous patients. While CTS-NT was described by some as having good staff retention rates compared with other NT health services, several care providers reported this negatively impacts on the consistency and quality of their relationships with Indigenous patients.

The amount of time spent in consultations with Indigenous patients was also reported to be longer due to the need to build rapport and consider cultural factors. Some clinicians offered patients the option to have their consults outside of the clinic on the grass and under a tree. This was seen by staff as a pivotal shift in the standard clinical approach and was regarded as fundamentally important when engaging with Indigenous patients.

*“Half of my consults are under the tree … not even inside the building. Actually, when the patient comes in, I first ask them, do you want me to come out or do you want to come in? If the patient’s preference is for me to come out, I am more than happy to be under the tree.” [Care provider, CP010].*

The physical environment of the CTS-NT was regarded by care providers as more welcoming for Indigenous patients than many other hospital settings, due to its size, location and outdoor amenities. However, the air-conditioning in the centre was attributed by care providers as a reason for Indigenous patients disliking and avoiding coming inside the service. The recent initiative of having a waiting room with painting facilities for patients to use while waiting for transport and appointments was described by care providers as being popular with some Indigenous patients.

## Conclusions

The participants in our study reported a range of barriers that impede access for Indigenous Australians to CTS in the NT, which span all five dimensions of accessibility. The key barriers arising from this study often traversed multiple dimensions of accessibility and were related to information and communication; cultural safety of care and involvement of Indigenous staff; flexibility in the provision of care; and the financial and social impact of attending cancer care. These barriers coalesced to make the experience of cancer diagnosis, treatment and follow-up care unduly confusing, fraught and difficult. Addressing these access barriers to CTS, particularly for those living outside major regional or urban areas in the NT, is likely to improve cancer outcomes for Indigenous cancer patients and their communities.

### Information and communication

Some Indigenous patients felt inadequately-informed about their cancer and the treatment, rendering them ill-prepared to make the difficult decisions they face about leaving family and community to uptake cancer care. As we have previously reported, one in four of these patients have moderate to high levels of unmet need related to a lack of information and communication about their cancer care, particularly around understanding the purpose and side-effects of cancer treatment [23]. Care providers similarly expressed concerns about the variable quality of communication with Indigenous patients and the impact this had on the timeliness, completeness, and quality of care provided. Some expressed difficulties in being able to appropriately deliver information without using difficult to understand medical jargon. In a previous Queensland study, care providers expressed similar concerns and identified a need for more culturally appropriate cancer information to assist care providers to communicate effectively and respectfully with Indigenous patients [20].

One patient in the current study suggested that training traditional healers to provide information on cancer and cancer treatment may empower communities to provide greater support to their members who require cancer care. A model of shared care involving traditional healers and Western physical and mental health practitioners has been demonstrated previously for remote Indigenous communities in Central Australia [26]. This model could be extended to include traditional healers providing health promotion and education to increase health literacy and access to health care; an approach that has been demonstrated outside Australia in rural and low resource settings [27].

### Cultural safety of care and involvement of Indigenous staff

In the current study, care providers felt misinformation and miscommunication resulted in patients’ mistrust of the CTS-NT. Shahid et al., found that mistrust in the health system associated with colonisation and racism was a key factor underpinning delays in cancer diagnosis in Indigenous people in Western Australia [28]. In this previous study, the authors argue that respectful and empathetic communication is necessary to re-build patients’ trust in CTS and ensure safe and timely access to health care services. To achieve this, they advocate for cultural safety training for care providers that fosters recognition of care providers’ own biases and increases their awareness of power imbalances and the ongoing impacts of colonisation. Evidence suggests that the current provision and completion of cultural safety training to health care staff may be inadequate, as a 2009 survey of Queensland CTS revealed that less than half of participating staff had participated in cultural safety, or similar, training at their work place [29].

The scarcity of Indigenous staff and care providers, together with a high staff turnover and variable access to language translation services, exacerbated the cultural disconnect between Indigenous patients and CTS-NT. Similar concerns have been identified for Indigenous cancer patients in Queensland [30], Western Australia [15] and Victoria [31]. Marcusson-Rababi et al. found that Indigenous women undergoing gynaecological cancer treatment in Queensland experienced a similar range of difficulties impacting their experience of cancer care, including: unsympathetic delivery of bad news, confusing terminology, language difference, and a lack of available interpreters [30]. Shahid et al. found that that providers commonly lacked an understanding of Aboriginal culture, and the socioeconomic conditions and life circumstances of Aboriginal families [15]. Ristevski et al. found that cancer care providers in Victoria has little understanding of the importance of cultural and family connections to Indigenous Australians undergoing cancer treatment [31]. The importance of having Indigenous health care providers working with Indigenous cancer patients has been identified in numerous studies [6, 15, 28, 32]. The value of ILOs in supporting Indigenous Australians with chronic illness has been reported in cardiovascular care [33].

### Flexibility in the provision of care

As has been reported previously [28], CTS care providers in the current study recognised that flexibility in the duration, timing, location, and number of attendees of the consultations, was imperative to ensuring culturally relevant and appropriate care for Indigenous cancer patients. A previous evaluation of telehealth using video consultations with Indigenous cancer patients found that patients were generally very satisfied with this model of care [34, 35]. Health workers in these studies attributed the value of telehealth to the easing of financial, travel, and time burdens on patients, and the opportunity it provides for community and family members to be involved in the specialist consultations [34, 35]. More broadly, a review of telehealth services for Indigenous Australians suggest this model of care has improved social and emotional wellbeing, clinical outcomes, and health service access [36]. The recent COVID-19 pandemic has necessitated adjustments in health care delivery, with a rapid shift towards a greater provision of telehealth [37]. It will be of great interest to assess in the coming months and years how this shift affected health care access and patient satisfaction and whether the shift was maintained beyond the pandemic.

### Financial and social impact of cancer care

Our study found that Indigenous Australians with cancer in the NT are plagued by transport, accommodation and financial difficulties, which add to the challenges of engaging with already demanding cancer treatment regimens. We have previously reported that worry about finances and accommodation when travelling away from home for cancer care were the two most commonly reported unmet supportive care needs among patients in this study [23]. Two previous studies identified similar difficulties for Indigenous patients in Queensland accessing transport and accommodation [19, 30]. Financial and affordability-related barriers have also been reported as high unmet needs in other Australian jurisdictions [23, 38]. While an expansion of telehealth services may reduce some need to travel for cancer care, some cancer care cannot be administered remotely and solutions to improve the availability of financial and other support services is warranted.

Care providers in this study were able to readily identify transport, accommodation, and financial distress as common barriers to accessibility of cancer care, however, it was unclear if CTS-NT was systematically screening patients to identify and support patients with such issues. Culturally-appropriate supportive care needs assessment tools have been developed and shown to greatly assist in identifying pertinent issues among patients who are engaged with the service, which can then enable timely referrals to appropriate services as required [38, 39]. Our study revealed that the responsibility for addressing unmet needs and accessibility issues at CTS-NT commonly falls to the ILO. Given the quantity and complexity of issues reported by patients in this study, the limited number and availability of ILO positions is wholly inadequate to meet the complex needs of this patient population.

Given the complexity of the barriers to accessing CTS that Indigenous Australians in the NT face, the solutions required to address the poorer cancer outcomes of this population are also complex and require coordination and cooperation across all levels of the health-system. A National Aboriginal and Torres Strait Islander Cancer Framework (Framework) [40] and the Optimal Care Pathway for Aboriginal and Torres Strait Islander People with Cancer (OCP) are available [41]. The implementation of the Framework and OCP should be undertaken in the NT in consultation with Indigenous stakeholders in this jurisdiction. In light of our findings and previous evidence, we make the following recommendations for all CTS in alignment with the Framework and OCP, that the following actions are taken:

- Stronger links are developed between tertiary CTS and community health services;
- Culturally-appropriate cancer resources and programs are developed and implemented in partnership with Indigenous communities to support doctor-patient communication, information provision and shared decision making;
- Cultural safety training is mandatory for all CTS staff;
- Strategies are developed and implemented to increase the number of clinical and allied Indigenous health staff at CTS, including ILOs;
- Support is provided for the expansion of telehealth programs within CTS;
- The Supportive Care Needs Assessment Tool for Indigenous peoples is used routinely in CTS for the timely identification of patient needs;
- Strategies are developed and implemented to link patients in need to available financial, travel, and accommodation that meets their needs;
- Flexibility of care provision in CTS, in terms of time, duration, location and size of consultations is investigated, developed and implemented.

Our recommendations for improving accessibility of CTS at the service-level can only be realised within the context of corresponding system-level improvements. Higher-level initiatives supported by governments are clearly needed to support the requisite changes, such as: facilitating stronger links between tertiary health services and regional and remote community health services; continued funding of existing telehealth programs; increasing tertiary training opportunities in cancer; and funding to support increased training, support and employment of Indigenous health workers in the NT.

#### Study limitations

There are some limitations with the design of this study to be considered when interpreting the study findings. The secondary data from patient interviews was predominantly from closed questions, which limits the scope for patients to fully communicate their experiences. Despite this limitation, the open-ended questions that were asked of patients did yield a broad range of barriers. Additionally, the interviewer of patients was a non-Indigenous researcher who was employed by CTS-NT, which may have discouraged patients from being critical of the service due to the perceived impact it could have on their care. As such, the barriers to access of care reported by patients may have been underestimated. Additionally, approximately five years has passed since the patient data was collected, which moderates the currency of the findings. Further, only the views of Indigenous patients who were attending CTS-NT were sought and we are missing insight from Indigenous people with cancer who do not attend the cancer centre. As such, it is likely that these findings underestimate the depth and complexity of the barriers that face the NT’s Indigenous population more broadly, particularly those who have no engagement with CTS.

Barriers to accessing CTS for Indigenous people in the NT must be overcome in order to address their significantly higher cancer burden. This study provides a valuable snapshot of the barriers facing this population across the five dimensions of health care access. Urgent action in addressing these issues is required at health professional, service, and systems level.

## Data Availability

The datasets used and/or analysed during the current study are not available due to privacy and confidentially restrictions.

## Appendix 1

### Questions asked of patients included in this analysis

1. Where did you stay when you needed to go to the hospital to see a cancer specialist or to get treatment?
2. Did you have to move away from family/community when you needed to go to a hospital to see a cancer specialist or to get treatment?
  [If yes, go to Q3; if no, go to Q13]
3. Why did you have to move away?
4. Do you know about the PATS subsidy?
5. When you go to hospital to see the cancer specialist or to get treatment, were you supported with accommodation?
6. At your accommodation, did you need a carer/escort?
7. How long were you away from your home?
8. Did you have any difficulties in getting to and from the airport, train or bus stations to get your accommodation or hospital appointment?
  - What were those difficulties?
9. How did you get to the grocery store to purchase food or personal items?
10. Did you have someone stay with you for your doctor’s appointments and treatments?
  - Were they are recognised ‘escort’ by the hospital?
11. How did you get from your accommodation to the hospital for your appointments?
12. Are there any items/questions or things you would like to tell us about regarding accessing specialists/hospitals that we have not asked you?
13. Did you have someone coming with you for your doctor’s appointments and treatments?
  - Were they are recognised ‘escort’ by the hospital?
14. How did you get from your home to the hospital for your appointments?
15. Did you have any difficulties in getting to and from the hospital for your appointments?
  - What were those difficulties?
16. Are there any items/questions or things you would like to tell us about regarding accessing specialists/hospitals that we have not asked you?

## Appendix 2

### Questions asked of care providers included in this analysis

1. Can you tell me a bit about your experiences generally with Indigenous cancer patients?
2. What do you see as the main issues facing Indigenous people with cancer?
3. Do you think that the cancer experiences of Indigenous people are different in any way to non-Indigenous people? If so in what way?
4. Are there any special considerations when treating Indigenous patients?
5. What sort of issues do Aboriginal people find most difficult about their treatments?
6. Are there specific issues around commencing or completing treatment? Are these different to non-Aboriginal patients? Are you aware of any cultural issues that come up for patients needing these treatments?
7. Could you describe the key issues that Aboriginal patients need support or assistance with?
8. What types of support services does the AWCCC provide for its patients?
9. Are you aware of whether Aboriginal patients use these support services? If yes, why or why/not?
10. Are there other support services that you feel would be beneficial for Aboriginal cancer patients?
11. Do you refer Aboriginal patients to use other support services? If so, which would be some of the most common ones you would refer to?

## Abbreviations

CTS: Cancer treatment service
CTS-NT: The Cancer treatment service in the Northern Territory where this study was undertaken
ILO: Indigenous Liaison Officer
NT: Northern Territory of Australia

## Footnotes

1 *Sorry Business* refers to a period of cultural practices and protocols associated with death.

2 Long grassing is a term commonly used in Darwin to describe homeless living or living rough.

3 An Australian Aboriginal term used in Arnhem Land to refer to a white person.
